# Genetic methylation and lymphoid malignancies: biomarkers of tumor progression and targeted therapy

**DOI:** 10.1186/2050-7771-1-24

**Published:** 2013-08-14

**Authors:** Xia Zhao, Wei Zhang, Li Wang, Wei-Li Zhao

**Affiliations:** 1 State Key Laboratory of Medical Genomics, Shanghai Institute of Hematology, Shanghai Rui Jin Hospital, Shanghai Jiao Tong University School of Medicine, 197 Rui Jin Er Road, Shanghai 200025, China; 2 Pôle de Recherches Sino-Français en Science du Vivant et Génomique, Laboratory of Molecular Pathology, Shanghai China

**Keywords:** Methylation, Leukemia, Lymphoma, Biomarker, Targeted therapy

## Abstract

Lymphoid malignancies, mainly including lymphocytic leukemia and lymphoma, are a group of heterogeneous diseases. Although the clinical outcome of patients has been significantly improved with current immuno-chemotherapy, definitive biomarkers remain to be investigated, particularly those reflecting the malignant behavior of tumor cells and those helpful for developing optimal targeted therapy. Recently, genome-wide analysis reveals that altered genetic methylations play an important role in tumor progression through regulation of multiple cellular transduction pathways. This review describes the pathogenetic effect of the aberrant genetic methylation in lymphoid malignancies, with special emphasis on potential therapeutic strategies targeting key signaling networks.

## Introduction

Lymphoid malignancies, mainly including lymphocytic leukemia and lymphoma, are a group of disorders that originate from neoplastic transformation of lymphocytes. Diseases vary in cells of origin, histologic appearances, molecular biology, clinical features and disease prognosis. Thus, searching for biomarkers closely related to tumor progression is critical to better understand the disease pathogenesis and to develop subsequent targeted therapy.

DNA methylation plays a pivotal role in transcriptional regulation. Aberrant promoter hypermethylation has been observed in cancer cells, which is responsible for the transcriptional silencing of tumor suppressor genes [1,2]. For example, tumor suppressor genes, such as *p15* (*CDKN2B*) [3], *p16* (*CDKN2A*) [4], *p57* (*CDKN1C*) [5], *p73* (*TP73*) [6], *SHP-1*[7] and *DAPK*[8], are frequently hypermethylated and related to tumor progression in lymphoid malignancies.

In this review, we described recent advances on alterations of genetic methylation profiling in lymphoid malignancies and highlighted their effects on specific signaling pathways involved in disease progression, which may be helpful in identifying strategies of targeted therapy.

### Part I: Genome-wide methylation in lymphoid malignancies

#### Acute lymphoblastic leukemia (ALL)

Acute lymphoblastic leukemia (ALL) is derived from malignant proliferation of immature lymphoblast cells committed to B- or T-cell lineage. They are the most common, and some of the most curable, tumors in children. ALL has lower prevalence in adults. However, patients usually have much poorer disease outcome.

In T-ALL, methylation pattern of 27 T-ALL-related genes were assessed by methylation specific-polymerase chain reaction (MS-PCR). These genes selected have crucial roles involved in cell cycle arrest (*p15*, *p16*, *p57* and *LATS-2*), apoptosis (*TMS1*, *APAF-1*, *DAPK* and *DIABLO*), differentiation regulation (*NES-1*), cell adhesion and metastasis process (*CDH1*, *CDH13*, *ADAMTS1* and *ADAMTS5*), tumor suppression (*LATS-1* and *PTEN*), WNT pathway (*DKK-3*, *WIF-1*, *sFRP-1*, *sFRP-2*, *sFRP-4* and *sFRP-5*), JAK-STAT pathway (*PTPN6 (SHP-1)*), p53 pathway (*p14*, *p73* and *PPP1R13B (ASPP-1)*), ubiquitination (*PARK-2*) and tyrosine kinase (*SYK*). Most of the T-ALL patients (44/50 cases) showed hypermethylation in at least one of these analyzed genes, particularly genes such as *NES-1*, *ADAMTS5*, *WIF-*1 and *sFRP-1* (Additional file 1) [9]. Patients were classified into two distinct groups according to the existence of CpG island methylator phenotype (CIMP): CIMP-positive, with three or more hypermethylated genes, and CIMP-negative, with two or less hypermethylated genes. Clinically, CIMP-positive patients presented significantly shorter disease-free survival (DFS) and overall survival (OS) than those of CIMP-negative counterparts.

A genome-wide study was performed on 19 *ETV6-RUNX1*-positive pediatric precursor B-ALL patients and revealed a unique DNA hypermethylation signature at diagnosis compared to remission. Associated genes were mostly implicated in cell fate commitment, gene regulation, and DNA binding where DNA methylation may have impaired gene function through transcriptional regulation. Interestingly, 15 hypermethylated genes were recurrent in B-ALL patients irrespective of the cytogenetic subtypes, including genes involved in cell cycle arrest (*MYOD1* and *BTG4*), cell development (*FOXE3, TCF3, PAX5* and *RAG1*), differentiation (*PTPRZ1, PPARG* and *IKZF1*) and WNT pathway (*sFRP-1*) [10].

Moreover, analysis of CpG methylation not only allowed T-ALL and B-ALL classification, but also distinguished subtypes among B-ALL patients. Methylation status of 416 methylation-involved or ALL-related genes was determined by methylation array in 401 patients with ALL [11]. Many of these genes were involved in key cellular functions like cell adhesion, apoptosis, proliferation and growth. Precursor B-ALL patients showed remarkably lower median methylation level than T-ALL patients. Methylation patterns clearly separated patients from the main cytogenetic subtypes of precursor B-ALL, such as high hyperdiploidy, t(12;21), 11q23, and t(1;19). Hypermethylation of *COBL, CPVL, EVC, LRP1B, PAX8, PCDHGA12* and *SPON2* were correlated with favorable prognosis.

Methylation status may also influence response to treatment in ALL. In a cohort study of initial diagnosed and relapsed matched B-ALL patients, genomic methylation level was distinctly higher in relapse than at newly diagnosis. A total of 905 genes were preferentially hypermethylated and 79 genes were hypomethylated. Integration analysis of methylation with gene expression profile and copy number abnormalities revealed six genes closely related to disease relapse. Among them, four genes (*CDKN2A, COL6A2, PTPRO* and *CSMD1*) were hypermethylated, down-regulated and focally deleted, and two genes (*NOTCH4* and *TOP1MT*) were hypomethylated, up-regulated and focally amplified [12]. Of these genes, *CDKN2A* is an important tumor suppressor which controls cell cycle by stabilizing p53. *PTPRO* encodes a receptor-type protein tyrosine phosphatase, which inhibits cell proliferation and induces apoptosis.

#### Chronic lymphocytic leukemia (CLL)

Chronic lymphocytic leukemia (CLL) is an indolent disease with clonal expansion of mature neoplastic B cells. Somatic mutation status of the immunoglobulin heavy-chain variable (*IGHV*) gene is an indicator of favorable prognosis [13].

Genome-wide methylation analysis was performed in 10 CLL patients. An average of 4.8% of CpGs analyzed was aberrantly methylated compared with normal neutrophils. One-hundred-and-seventy-three genes were differentially methylated [14]. This study identified a hypermethylated gene, *GRM7*, which inhibited cAMP and induced cell apoptosis. Another methylation array covering 14495 genes was performed in 23 CLL cases. Significant difference in methylation pattern was observed between *IGHV*-unmutated and *IGHV*-mutated CLL subgroups, with 64 genes differentially methylated. Hypermethylated tumor suppressor genes (*ABI3, SCGB2A1, VHL, GPX3, IGSF4* and *SERPIND5*) were identified in unmutated CLL. In mutated cases, hypermethylation genes were involved in cell proliferation and NF-κB pathway (*ADORA3, AIRE, CARD15, LOC340061, UNC5CL* and *LDOC1*) and MAPK pathway (*PRF1, FABP7*) [15].

Methylation profiling is also indicative in CLL prognosis prediction. Genome-wide analysis of methylated CpG amplification, coupled with promoter microarray, identified 22 of 280 aberrantly methylated genes in 78 CLL patients, compared to normal B cells from 10 healthy volunteers. These genes were enriched in cellular functions, including cell growth and differentiation, tissue and organ development, tissue morphology, cancer, cell death and cell cycle regulation [16]. Twenty-seven genes were mapped to the neighboring genetic region of *TP53* in chromosome 17. Among these genes, *PRIMA1, TFAP4, SIRT2* and *TP53INP2* were previously reported to functionally interact with p53. Bisulfite pyrosequencing further confirmed hypermethylation status of 19 candidate genes (*SOX11, DLX1, FAM62C, SOX14, RSPO1, ADCY5, HAND2, SPOCK, ING1, PRIMA1, BCL11B, LTBP2, NR2F2, GALGT2, LHX1, DLX4, KLK10, TFAP2* and *APP*). Survival analysis showed that *LINE, APP, SALL1* and *PRIMA1* were correlated with shorter OS.

#### Malignant lymphoma

Malignant lymphoma mainly includes non-Hodgkin’s lymphoma (NHL) and Hodgkin’s lymphoma (HL). Its incidence is increasing and now ranges among the tenth most frequent cancers worldwide.

#### Diffuse large B-cell lymphoma (DLBCL)

Diffuse large B-cell lymphoma (DLBCL) is one of the most common NHL. Two biologically distinct subtypes are identified by gene expression profiling: germinal center B-cell-like (GCB) DLBCL and activated B-cell-like (ABC) DLBCL [17].

Genome-wide methylation was analyzed in 24 GCB-DLBCL and 21 ABC-DLBCL patients. The CpGs of 12 genes showed a hypermethylation pattern in both DLBCL subtypes, including genes involved in cell cycle arrest (*CDKN1C* and *MYOD1*), apoptosis (*GDNF*), Rho pathway (*DLC1*), transcription factors (*AR, GATA4, NEUROD1, ONECUT2* and *TFAP2A*), receptor proteins (*DRD2* and *GRIN2B*) and metabolism process (*MTHFR*). Methylation status of genes *FLJ21062* (*C7orf63*), *ONECUT2* and *GNMT* differed between GCB-DLBCL and ABC-DLBCL. In addition, *FLJ21062, BNIP3, MGMT, RBP1, GATA4, IGSF4* and *CRABP1* showed significantly increased levels of DNA methylation with decreased gene expression [18].

Another study measured gene methylation status in 69 DLBCL patients treated with Rituximab combined with CHOP regimen (cyclophosphamide, hydroxydaunorubicin, vincristine, and prednisone). Supervised analysis identified 263 differentially methylated genes between GCB-DLBCL and ABC-DLBCL subtypes. These genes were mostly enriched in antigen processing-presentation pathway, cytokine and inflammatory pathway. Integrated data with expression profile further confirmed 16 genes (*LANCL1, KCNK12, SORL1, CXorf57, SOX9, KIAA0746, ASPHD2, ARHGAP17, IKZF1, PMM2, IL12A, JDP2, PAK1, GALNS, FGD2* and *LYAR*) that distinguished these two subtypes with 92%-98% accuracy [19]. Some of these genes were previously recognized in signaling pathways, such as JAK-STAT (*IL12A*) and Rho pathway (*ARHGAP17, PAK1* and *FDG2*).

#### Mantle cell lymphoma (MCL)

Mantle cell lymphoma (MCL) is an aggressive B-NHL that arises from naïve B cells in the mantle zone of the lymph nodes. It is characterized by t(11;14)(q13;q32) translocation and subsequent overexpression of *CCND1*.

Methylation study in MCL cell lines found 331 differentially expressed genes mapped to autosomal chromosomes. Pathway analysis revealed that top cellular functions represented cell death, cell cycle, cellular growth and proliferation. MassARRAY assay of 25 candidate genes in seven MCL cell lines and normal B cells showed 20 genes hypermethylated in more than one MCL cell line, encompassing cell differentiation (*SOX9*), cell adhesion (*CDH1*), cell cycle (*GOS2*) and apoptosis (*LGALS3*), p53 pathway (*CDC14B*) and PI3K pathway (*THEM4*), transcription factors (*AHR, HOXA9, NR2F2, FOXC1, TWIST1*) and others with unknown functions (*ROBO1, NPTX2, CYB1B1, GPX3, MAL, PAX6, PTPRG1, TFPI2*). Among them, seven genes, whose methylation was reported in different tumor models, appeared to be frequently methylated in primary cells of 38 MCL patients (*SOX9*, *HOXA9*, *AHR*, *NR2F2*, *ROBO1*, *NPTX2* and *CDH1*). MCL patients with two or more methylated genes had higher proliferation index Ki-67, increased number of chromosomal abnormalities and shorter OS than those with none or only one methylated gene. Methylation of tumor suppressor genes *SOX9* and *HOXA9* were also associated with the above-mentioned clinicopathological parameters and poor disease outcome in MCL [20].

Another genome-wide methylation analysis was performed in primary MCL samples and found significant aberrations in promoter methylation pattern compared with normal B cells. DNA methylation was quantified for over 14000 gene promoter regions. Integration of methylation and expression profiling data revealed 4 hypermethylated genes (*CDKN2B, HOXD8, MLF-1* and *PCDH8*) and 4 hypomethylated genes (*CD37, HDAC1, NOTCH1* and *CDK5*). Among these genes, *CDKN2B* and *CDK5* are involved in cell cycle control. *HDAC1* and *NOTCH1* are known targets for treating lymphoid malignancies. *HOXD8, MLF-1* and *PCDH8* are implicated in multiple tumor types. *CD37* belongs to tetraspanin transmembrane family and is expressed mainly in B cells [21].

#### Hodgkin’s lymphoma (HL)

Screening of methylated genes was performed in HL KM-H2 cell line by microarray analysis before and after treatment with 5-aza-2’-deoxycitidine. Thirty tumor suppressive genes were identified, including genes in cellular adhesion (*CADM1 (IGSF4), CD44* and *THBS1*)*,* growth arrest (*GADD45*), p53 pathway (*ZMAT3*) and two transcription factors (*IRF7* and *KLF6*). Among them, *CADM1* was further confirmed methylated and down-regulated in primary HL cells. Restoration of *CADM1* expression in HL cells decreased cell survival and increased their sensitivity to apoptosis, demonstrating that *IGSF4* silencing by CpG methylation inhibited apoptosis in Reed-Sternberg cells, which was an important process in HL pathogenesis [22].

Another genome-wide methylation study of 13088 genes compared five HL cell lines (L-1236, L-428, KM-H2, HDLM-2 and U-HO1) to normal B cells and 20 germinal center derived B-cell lymphoma (gcdBCL). HL and gcdBCL showed 329 commonly hypermethylated genes, mainly involved in development and morphogenesis, WNT pathway and regulation of adenylated cyclase activity. Two-hundred-and-nine genes were distinctly hypermethylated in HL cell lines, compared to gcdBCL or normal B cells. Gene Ontology analysis indicated that genes were enriched for positive regulation of B-cell activation and T-cell differentiation, suggesting that hypermethylated genes in HL targeted the B-cell program [23].

### PART II: Signaling pathways involved in alterations of methylation status

**Figure 1:**
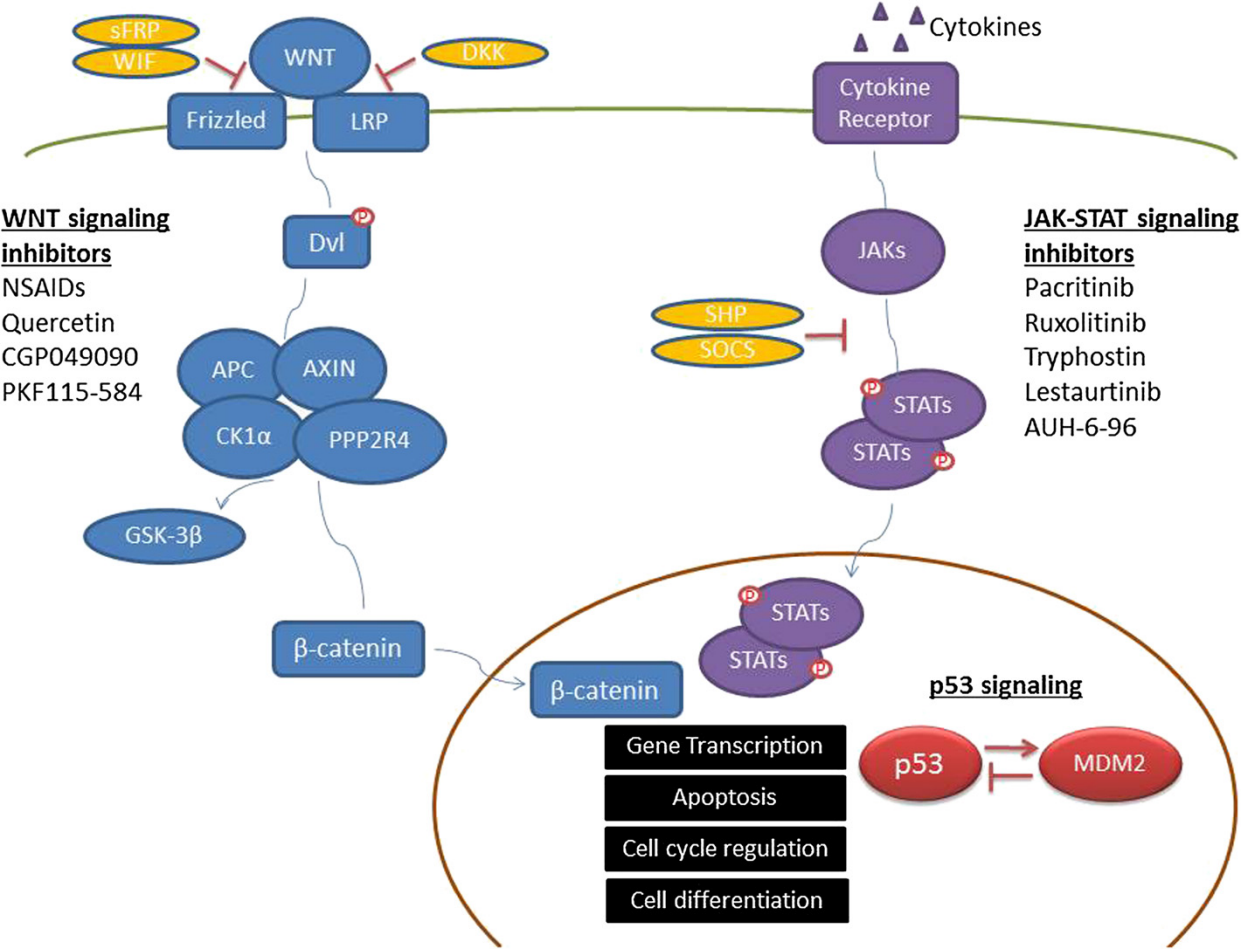
**Key signaling pathways involved in genetic methylation of lymphoid malignancies.** In WNT pathway, WNT binds to Frizzled and LRP to phosphorylate Dvl and downstream degrades complex containing APC, AXIN, CK1α, PPP2R4 and GSK-3β. β-catenin is then released and translocated into the nucleus, activating target gene expression. In JAK-STAT pathway, cytokines bind to transmembrane receptors and activate JAKs, who then activate transcription factors STATs. STATs then translocate into the nucleus and initiate target gene transcription. P53 is an important tumor suppressor, and functions through both transcription dependent and independent ways. MDM2 is the main negative regulator of p53. In lymphoid malignancies, important negative regulatory genes (indicated in yellow) are found hypermethylated and down-regulated, resulting in aberrantly activated signaling in tumor cells. Targeted therapies for specific WNT and JAK-STAT signaling pathways under preclinical and clinical evaluations are listed.

Epigenetic profiling showed different genomic DNA methylation patterns among diseases. Several signaling pathways, such as WNT pathway, JAK-STAT pathway and p53 pathway, are recurrently involved in lymphoid malignancies (Figure 1).

#### WNT pathway

WNT pathway is essential in hematopoiesis and plays an important role in controlling cell proliferation, differentiation and survival [24]. *WNT* encodes a family of secreted cysteine-rich glycoproteins. The ligand receptor of WNT contains transmembrane protein Frizzled and LPR5/6. The main downstream target of WNT pathway is β-catenin. In the absence of WNT binding to its receptor, β-catenin is stabilized and bound to E-cadherin. The excess β-catenin protein is phosphorylated and degraded by destruction complex containing APC, AXIN, CK1α, PPP2R4 and GSK-3β. Upon activation, the WNT ligand binds to its membrane receptors and cytoplasmic protein Dvl is accordingly recruited and activated by phosphorylation. Activated Dvl induces dissociation and subsequent inhibition of GSK-3β from the complex. β-catenin is then released and translocated into the nucleus, where it up-regulates target gene expression. There are also several antagonist proteins of WNT-Frizzled interaction, including DKK, WIF and sFRP family members.

Increasing evidence showed dysregulation of WNT signaling in lymphoid malignancies. Pediatric precursor B-ALLs present hypermethylation of WNT inhibitors (*WIF-1, PTPRO, sFRP2, sFRP4, sFRP5, FZD10, DKK2* and *DKK3*), β-catenin/TCF/LEF complex inhibitors (*APC* and *WT1*), cadherin genes (*CDH1, CDH11* and *CD13*) and SOX genes (*SOX2, SOX3, SOX8, SOX9, SOX14* and *SOX21*). Moreover, in Ph-positive ALLs, the silencing of *sFRP1*, *sFRP2*, *sFRP4*, *sFRP5*, *WIF1*, *DKK3*, *HDPR1* and *WNT5A* by methylation predicts longer DFS and OS [25]. Primary CLL samples show hypermethylation pattern of *sFRP* gene family, especially *sFRP1* and *sFRP4*, [26]. Other WNT inhibitors, such as *WIF1* and *DKK3*, are also methylated in CLL [27].

#### JAK-STAT pathway

JAK-STAT pathway plays an essential role in the transduction of extracellular cytokine signals into intracellular process during hematopoietic ontogeny. The JAK family contains four cytoplasmic tyrosine kinases (JAK1-3 and TYK2) which are activated after cytokine receptor activation. Downstream of JAKs activation is phosphorylation of STATs transcription factor family (STAT1-4, STAT5A, STAT5B and STAT6). The phosphorylated STATs form homo- or heterodimers and translocate into the nucleus, initiating target gene transcription [28]. Constitutive STAT activation leads to increased cell proliferation, tumorigenesis and decreased apoptosis.

*SHP1* is an important tumor suppressor gene which blocks the JAK-STAT pathway by dephosphorylating the receptors and receptor-associated kinases. Frequent methylation of *SHP1* was observed in MCL, as compared to normal B cells [29]. In another study, *SHP1* methylation was observed in 100% NHL and 94% leukemia patients, with mRNA expression accordingly decreased [30]. This study also found recurrent methylation of *SOCS1* gene, inhibitor of the JAK-STAT pathway through blocking JAK activation, in about 30% of NHL and leukemia patients.

#### P53 pathway

*TP53* is one of the most frequently altered tumor suppressor genes in cancer. The encoded p53 protein, which is ubiquitously expressed in tissue, keeps genome stability under stress, and is involved in multiple cellular activities such as development, differentiation, aging and disease [31]. In normal state, p53 is inhibited and degraded by MDM2. When activated, p53 exerts its function through transcription-dependent and -independent activities.

Methylation in promoter CpG or in CCWGG motif of *TP53* was observed in 32% of ALL patients and correlated with decreased mRNA expression [32]. In CLL and DLBCL cases, methylation of *TP53* was also observed, but with less methylation rate (18.5% and 3.7%, respectively) [33,34]. A recent study analyzed 24 genes involved in the p53 pathway in six ALL cell lines and found 13 genes to be aberrantly hypermethylated. These genes are involved in the regulation of p53-dependent apoptosis (*DBC1, POU4F2, AMID, APAF1, ASPP1, TP73, NOXA, has-miR-34b* and *has-miR-34c,*), cell cycle regulation (*POU4F1* and *CDKN1C*) and regulation of p53 itself (*LATS2* and *DAPK1*). Further confirmation analysis on independent cohort of 200 ALL cases showed that 78% of them present methylation in at least one of these 13 genes. These patients are characterized by lower complete remission (CR) rate (84% versus 91% in non-methylated group), higher relapse rate (46% versus 21%) and higher mortality rate (56% versus 19%). Survival analysis further indicated that hypermethylation of these genes is an independent prognostic factor both for DFS and OS [35].

### PART III: Targeted therapy on genetic methylation and involved signaling pathways

Different from genetic abnormalities, epigenetic aberrations are possibly reversible. Great efforts have been made on the development of epigenetic modulators, mainly focusing on the inhibition of DNA methyltransferase (DNMT) and histone deacetylase (HDAC). Also, specific targeted therapies on involved signaling pathways are under active preclinical investigation.

#### DNMT inhibitors

DNMT inhibitors are small molecules that effectively inhibit DNA methylation. 5’-azacytidine (Azacytidine, Vidaza) and 5-aza-2’-deoxycitidine (Decitabine, Dacogen) have been approved by Food and Drug Administration (FDA) for treating hematological malignancies [36].

Successful treatment with 5’-azacytidine was first reported in a 74-year-old female patient with myelodysplastic syndrome transformed pre-B ALL. At the time of transformation, bone marrow examination demonstrated pre-B blasts with del(20)(q11.2). The patient received 5’-azacytidine monotherapy (75mg/m^2^ subcutaneously, for 7 consecutive days every 28 days, 6 cycles) and attained CR for over 6 months [37]. Induction of 5-aza-2’-deoxycitidine in childhood refractory ALL was also reported [38]. A four-year-old girl with relapsed B-ALL from hematopoietic stem cell transplantation (HSCT) received 5-aza-2’-deoxycitidine (15mg/m^2^ i.v., three times a day for 3 days) combined with dexamethasone. The patient achieved CR and received a second allogeneic HSCT.

A Phase II trial of 5’-azacytidine was carried out in nine patients with recurrent fludarabine-refractory CLL. All cases received 1 to 6 cycles of 5’-azacytidine (75 mg/m^2^ subcutaneously, for 7 consecutive days every 3 to 8 weeks). Although this trial was discontinued because of lack of response and treatment tolerance, one patient responded to treatment after four cycles with clinical improvement and was alive at last follow-up [39]. Another Phase I trial of 5-aza-2’-deoxycitidine in 16 relapsed/refractory CLL and four NHL patients aimed to determine the minimum effective dose of 5-aza-2’-deoxycitidine. Three different escalating dose schedules were examined. All patients tolerated well the first dose group (10 mg/m^2^/day for 10 days). In the other two dose groups (15 mg/m^2^/day for 10 days or 15 mg/m^2^/day for 5 days), dose-limiting toxicity occurred in 67% (4 out of 6 patients) and 50% (4 out of 8 patients) patients, respectively. Main adverse effects consisted of grade 3 to 4 neutropenia, thrombocytopenia and hyperbilirubinemia. Among twenty patients treated, eight had stable disease (SD) [40]. *In vitro*, 5’-azacytidine also showed cytotoxic activity in primary samples of high-risk (11q-/17p-) and low-risk (13q-) CLL [41].

#### HDAC inhibitors

HDAC inhibitors can open chromatin status and suppress histone deacetylase, thus inducing transcriptional reactivation of silenced genes. Major HDAC inhibitors approved by FDA include Romidepsin (Depsipeptide), Panobinostat and Suberoylanilide hydroxamic acid (SAHA, vorinostat).

Romidepsin was used in a Phase I clinical trial for treating 10 CLL patients. 13 mg/m^2^ Romidepsin was given intravenously on days 1, 8 and 15. No patient met the response criteria. However, seven had improved peripheral leukocyte counts and one had lymphadenopathy reduction [42]. Another Phase II study enrolled 25 patients with relapsed/refractory HL treated with 200 mg SAHA (twice daily 14 days every 3 weeks or 3 days per week). One patient achieved partial response (PR) and seven other patients had SD over 1 year [43]. Panobinostat was also effective in treating refractory HL. In a cohort of 129 HL patients relapse/refractory to HSCT, Panobinostat (40 mg 3 times per week) was well tolerated and achieved an overall response rate (ORR) of 27%, including 23% PR and 4% CR. Median PFS was 6.1 months and estimated 1-year OS rate was 78% [44].

*In vitro*, ALLs with MLL-rearrangement present particular hypomethylation pattern of proto-oncogenes, such as *MYC*, *SET*, *RUNX1*, *RAN*. HDAC inhibitors (Romidepsin, Panobinostat, SAHA, and Valproic acid) can reverse such unfavorable epigenetic pattern and effectively induce leukemic cell death [45]. Moreover, HDAC inhibitors synergistically interacted with 5-aza-2’-deoxycitidine in ALL, DLBCL and MCL cell lines [21,46,47].

#### Targeting WNT signaling pathway

Targeted therapies circumventing the WNT signaling pathway were in the preclinical stage. Their functions include inhibition of WNT-mediated transcription and inactivation of WNT target genes.

Non-steroid anti-inflammatory drugs (NSAIDs) inhibit the WNT signaling pathway through preventing β-catenin transcription complex formation. In T-cell leukemia Jurkat cell line, para-nitric oxide-donating acetylsalicylic acid (para-NO-ASA) induces β-catenin degradation, along with an increase of caspase-3 expression and induction of apoptosis [48]. Similar apoptosis-inducing effect is observed in primary CLL cells and in a xenograft murine model, while sparing normal hematopoiesis *in vitro*[49].

Quercetin is an inhibitor of β-catenin through GSK-3β inactivation [50]. In cell lines of T-ALL, DLBCL and MCL [51], quercetin inhibits WNT signaling and induces cell growth arrest and apoptosis. Importantly, *CCND1*, as well as anti-apoptotic *BCL-XL* and *BCL-2*, are significantly down-regulated after quercetin treatment [52].

Small molecule inhibitors of WNT cascade (CGP049090 and PKF115-584) were tested in CLL. These inhibitors efficiently induce CLL cell apoptosis, while normal B cells are not affected. Both agents suppressed tumor growth in a JVM-3 subcutaneous xenograft murine model [53].

Although not tested in lymphoid malignancies, several antibodies have been proved to inhibit proliferation and induce apoptosis in solid tumors, including antagonists of WNT [54,55] and of WNT receptors [56,57]. In addition, peptide mimetics are identified in modulating the WNT signaling. For example, the sFRP1-derived peptides inhibit colorectal cancer xenograft formation in mice [58].

#### Targeting JAK-STAT signaling pathway

Pacritinib (SB1518) is an inhibitor of JAK2. A Phase I trial on Pacritinib in 34 relapsed/refractory HL or NHL patients show well tolerance. Three PRs are observed at a dose of 300 mg/day. Fifteen patients achieved SD, 13 of which had 4%-46% tumor reductions. Most common adverse events are gastrointestinal toxicities [59].

Ruxolitinib is a JAK1/JAK2 inhibitor and a FDA approved drug for treatment of myeloproliferative neoplasm. Recently its anti-proliferative effect was reported in cell lines of HL and primary mediastinal B-cell lymphoma. Expression of anti-apoptotic gene *BCL-XL* and *MCL-1* decreased in a dose-dependent manner [60]. Also *in vitro*, Pacritinib induces cell apoptosis, cell cycle arrest and inhibits proliferation in lymphoid cell lines, including Jurkat and MOLT-4 [61]. Other JAK2 inhibitors include Tryphostin (AG-490) and Lestaurtinib. Tryphostin enhances the cytotoxicity effect of tumor necrosis factor-related apoptosis-inducing ligand (TRAIL) in primary T-ALL cells and in Jurkat and SUPT1 T cell lines [62]. Lestaurtinib induces dose-dependent cell growth inhibition and apoptosis in five HL cell lines derived from refractory patients and primary cells obtained from four HL patients [63].

AUH-6-96 is firstly identified in *Drosophila*. In HL cell line L540, AUH-6-96 treatment abrogates the signaling pathway by reducing the level of JAK3 and STAT3 phosphorylation, thus down-regulating *BCL-XL* expression. Cell viability assay showed that this drug selectively induces apoptosis in the cancer cell line, while normal cells are not affected [64].

## Conclusions

Genetic methylation plays an important role in tumor transformation and progression in lymphoid malignancies. Accumulating data of DNA methylation study not only lead to disease classification and risk stratification, but also revealed multiple signaling pathways for targeted therapy development. Although preclinical and clinical investigations indicated their beneficial effects on patients, further study should be focused on the bio-therapeutic agents targeting the specific methylated genes and the methylation status of involved signaling pathways.

## Abbreviations

ALL: Acute lymphoblastic leukemia; MS-PCR: Methylation specific-polymerase chain reaction; CIMP: CpG island methylator phenotype; DFS: Disease-free survival; OS: Overall survival; CLL: Chronic lymphocytic leukemia; IGHV: Immunoglobulin heavy-chain variable; NHL: Non-Hodgkin’s lymphoma; HL: Hodgkin’s lymphoma; DLBCL: Diffuse large B-cell lymphoma; GCB: Germinal center B-cell-like; ABC: Activated B-cell-like; MCL: Mantle cell lymphoma; gcdBCL: Germinal center derived B-cell lymphoma; CR: Complete remission; DNMT: DNA methyltransferase; HDAC: Histone deacetylase; FDA: Food and Drug Administration; HSCT: Hematopoietic stem cell transplantation; SD: Stable disease; PR: Partial response; ORR: Overall response rate; NSAIDs: Non-steroid anti-inflammatory drugs; para-NO-ASA: Para-nitric oxide-donating acetylsalicylic acid; TRAIL: Tumor necrosis factor-related apoptosis-inducing ligand.

## Competing interests

The authors declared that they have no competing interest.

## Authors’ contributions

WLZ had the idea of conducting this review, XZ and WLZ wrote the manuscript, WZ and LW gave suggestions on modifications. All authors read and approved the final manuscript.

## Supplementary Material

Additional file 1 Identified gene hypermethylation in lymphoid malignancies Click here for file

## References

[B1] Chen W Cooper TK Zahnow CA Overholtzer M Zhao Z Ladanyi M Karp JE Gokgoz N Wunder JS Andrulis IL Levine AJ Mankowski JL Baylin SB Epigenetic and genetic loss of Hic1 function accentuates the role of p53 in tumorigenesis Cancer Cell 2004 6 4 387 398 10.1016/j.ccr.2004.08.030 15488761

[B2] Hoffmann MJ Schulz WA Causes and consequences of DNA hypomethylation in human cancer Biochem Cell Biol 2005 83 3 296 321 10.1139/o05-036 15959557

[B3] Hutter G Scheubner M Zimmermann Y Kalla J Katzenberger T Hubler K Roth S Hiddemann W Ott G Dreyling M Differential effect of epigenetic alterations and genomic deletions of CDK inhibitors [p16(INK4a), p15(INK4b), p14(ARF)] in mantle cell lymphoma Genes Chromosomes Cancer 2006 45 2 203 210 10.1002/gcc.20277 16258956

[B4] Drexler HG Review of alterations of the cyclin-dependent kinase inhibitor INK4 family genes p15, p16, p18 and p19 in human leukemia-lymphoma cells Leukemia 1998 12 6 845 859 10.1038/sj.leu.2401043 9639410

[B5] Hagiwara K Li Y Kinoshita T Kunishma S Ohashi H Hotta T Nagai H Aberrant DNA methylation of the p57KIP2 gene is a sensitive biomarker for detecting minimal residual disease in diffuse large B cell lymphoma Leuk Res 2010 34 1 50 54 10.1016/j.leukres.2009.06.028 19616848

[B6] Corn PG Kuerbitz SJ van Noesel MM Esteller M Compitello N Baylin SB Herman JG Transcriptional silencing of the p73 gene in acute lymphoblastic leukemia and Burkitt’s lymphoma is associated with 5’ CpG island methylation Cancer Res 1999 59 14 3352 3356 10416592

[B7] Koyama M Oka T Ouchida M Nakatani Y Nishiuchi R Yoshino T Hayashi K Akagi T Seino Y Activated proliferation of B-cell lymphomas/leukemias with the SHP1 gene silencing by aberrant CpG methylation Lab Invest 2003 83 12 1849 1858 10.1097/01.LAB.0000106503.65258.2B 14691303

[B8] Esteller M Profiling aberrant DNA methylation in hematologic neoplasms: a view from the tip of the iceberg Clin Immunol 2003 109 1 80 88 10.1016/S1521-6616(03)00208-0 14585279

[B9] Roman-Gomez J Jimenez-Velasco A Agirre X Prosper F Heiniger A Torres A Lack of CpG island methylator phenotype defines a clinical subtype of T-cell acute lymphoblastic leukemia associated with good prognosis J Clin Oncol 2005 23 28 7043 7049 10.1200/JCO.2005.01.4944 16192589

[B10] Wong NC Ashley D Chatterton Z Parkinson-Bates M Ng HK Halemba MS Kowalczyk A Bedo J Wang Q Bell K Algar E Craig JM Saffery R A distinct DNA methylation signature defines pediatric pre-B cell acute lymphoblastic leukemia Epigenetics 2012 7 6 535 541 10.4161/epi.20193 22531296

[B11] Milani L Lundmark A Kiialainen A Nordlund J Flaegstad T Forestier E Heyman M Jonmundsson G Kanerva J Schmiegelow K Soderhall S Gustafsson MG Lonnerholm G Syvanen AC DNA methylation for subtype classification and prediction of treatment outcome in patients with childhood acute lymphoblastic leukemia Blood 2010 115 6 1214 1225 10.1182/blood-2009-04-214668 19965625

[B12] Hogan LE Meyer JA Yang J Wang J Wong N Yang W Condos G Hunger SP Raetz E Saffery R Relling MV Bhojwani D Morrison DJ Carroll WL Integrated genomic analysis of relapsed childhood acute lymphoblastic leukemia reveals therapeutic strategies Blood 2011 118 19 5218 5226 10.1182/blood-2011-04-345595 21921043 PMC3217405

[B13] Hamblin TJ Davis Z Gardiner A Oscier DG Stevenson FK Unmutated Ig V(H) genes are associated with a more aggressive form of chronic lymphocytic leukemia Blood 1999 94 6 1848 1854 10477713

[B14] Rush LJ Raval A Funchain P Johnson AJ Smith L Lucas DM Bembea M Liu TH Heerema NA Rassenti L Liyanarachchi S Davuluri R Byrd JC Plass C Epigenetic profiling in chronic lymphocytic leukemia reveals novel methylation targets Cancer Res 2004 64 7 2424 2433 10.1158/0008-5472.CAN-03-2870 15059895

[B15] Kanduri M Cahill N Goransson H Enstrom C Ryan F Isaksson A Rosenquist R Differential genome-wide array-based methylation profiles in prognostic subsets of chronic lymphocytic leukemia Blood 2010 115 2 296 305 10.1182/blood-2009-07-232868 19897574

[B16] Tong WG Wierda WG Lin E Kuang SQ Bekele BN Estrov Z Wei Y Yang H Keating MJ Garcia-Manero G Genome-wide DNA methylation profiling of chronic lymphocytic leukemia allows identification of epigenetically repressed molecular pathways with clinical impact Epigenetics 2010 5 6 499 508 10.4161/epi.5.6.12179 20484983 PMC3322493

[B17] Alizadeh AA Eisen MB Davis REMAC Lossos IS Rosenwald A Boldrick JC Sabet H Tran TYUX Powell JI Yang L Marti GE Moore T Hudson J jr Lu L Lewis DB Tibshirani R Sherlock G Chan WC Greiner TC Weisenburger DD Armitage JO Warnke R Levy R Wilson W Grever MR Byrd JC Botstein D Brown PO Distinct types of diffuse large B-cell lymphoma identified by gene expression profiling Nature 2000 403 6769 503 511 10.1038/35000501 10676951

[B18] Pike BL Greiner TC Wang X Weisenburger DD Hsu YH Renaud G Wolfsberg TG Kim M Weisenberger DJ Siegmund KD Ye W Groshen S Mehrian-Shai R Delabie J Chan WC Laird PW Hacia JG DNA methylation profiles in diffuse large B-cell lymphoma and their relationship to gene expression status Leukemia 2008 22 5 1035 1043 10.1038/leu.2008.18 18288132 PMC2654231

[B19] Shaknovich R Geng H Johnson NA Tsikitas L Cerchietti L Greally JM Gascoyne RD Elemento O Melnick A DNA methylation signatures define molecular subtypes of diffuse large B-cell lymphoma Blood 2010 116 20 e81 e89 10.1182/blood-2010-05-285320 20610814 PMC2993635

[B20] Enjuanes A Fernandez V Hernandez L Navarro A Bea S Pinyol M Lopez-Guillermo A Rosenwald A Ott G Campo E Jares P Identification of methylated genes associated with aggressive clinicopathological features in mantle cell lymphoma PloS One 2011 6 5 e19736 10.1371/journal.pone.0019736 21603610 PMC3095614

[B21] Leshchenko VV Kuo PY Shaknovich R Yang DT Gellen T Petrich A Yu Y Remache Y Weniger MA Rafiq S Suh KS Goy A Wilson W Verma A Braunschweig I Muthusamy N Kahl BS Byrd JC Wiestner A Melnick A Parekh S Genomewide DNA methylation analysis reveals novel targets for drug development in mantle cell lymphoma Blood 2010 116 7 1025 1034 10.1182/blood-2009-12-257485 20427703 PMC2938124

[B22] Murray PG Fan Y Davies G Ying J Geng H Ng KM Li H Gao Z Wei W Bose S Anderton J Kapatai G Reynolds G Ito A Marafioti T Woodman CB Ambinder R Tao Q Epigenetic silencing of a proapoptotic cell adhesion molecule, the immunoglobulin superfamily member IGSF4, by promoter CpG methylation protects Hodgkin lymphoma cells from apoptosis Am J Pathol 2010 177 3 1480 1490 10.2353/ajpath.2010.100052 20709797 PMC2928979

[B23] Ammerpohl O Haake A Pellissery S Giefing M Richter J Balint B Kulis M Le J Bibikova M Drexler HG Seifert M Shaknovic R Korn B Kuppers R Martin-Subero JI Siebert R Array-based DNA methylation analysis in classical Hodgkin lymphoma reveals new insights into the mechanisms underlying silencing of B cell-specific genes Leukemia 2012 26 1 185 188 10.1038/leu.2011.194 21818115

[B24] Clevers H Nusse R Wnt/beta-catenin signaling and disease Cell 2012 149 6 1192 1205 10.1016/j.cell.2012.05.012 22682243

[B25] Martin V Agirre X Jimenez-Velasco A Jose-Eneriz ES Cordeu L Garate L Vilas-Zornoza A Castillejo JA Heiniger A Prosper F Torres A Roman-Gomez J Methylation status of Wnt signaling pathway genes affects the clinical outcome of Philadelphia-positive acute lymphoblastic leukemia Cancer Sci 2008 99 9 1865 1868 10.1111/j.1349-7006.2008.00884.x 18549404 PMC11159008

[B26] Liu TH Raval A Chen SS Matkovic JJ Byrd JC Plass C CpG island methylation and expression of the secreted frizzled-related protein gene family in chronic lymphocytic leukemia Cancer Res 2006 66 2 653 658 10.1158/0008-5472.CAN-05-3712 16423993

[B27] Chim CS Pang R Liang R Epigenetic dysregulation of the Wnt signalling pathway in chronic lymphocytic leukaemia J Clin Pathol 2008 61 11 1214 1219 10.1136/jcp.2008.060152 18765431

[B28] Chen E Staudt LM Green AR Janus kinase deregulation in leukemia and lymphoma Immunity 2012 36 4 529 541 10.1016/j.immuni.2012.03.017 22520846 PMC7480953

[B29] Chim CS Wong KY Loong F Srivastava G SOCS1 and SHP1 hypermethylation in mantle cell lymphoma and follicular lymphoma: implications for epigenetic activation of the Jak/STAT pathway Leukemia 2004 18 2 356 358 10.1038/sj.leu.2403216 14614518

[B30] Reddy J Shivapurkar N Takahashi T Parikh G Stastny V Echebiri C Crumrine K Zochbauer-Muller S Drach J Zheng Y Feng Z Kroft SH McKenna RW Gazdar AF Differential methylation of genes that regulate cytokine signaling in lymphoid and hematopoietic tumors Oncogene 2005 24 4 732 736 10.1038/sj.onc.1208032 15580314

[B31] Xu-Monette ZY Medeiros LJ Li Y Orlowski RZ Andreeff M Bueso-Ramos CE Greiner TC McDonnell TJ Young KH Dysfunction of the TP53 tumor suppressor gene in lymphoid malignancies Blood 2012 119 16 3668 3683 10.1182/blood-2011-11-366062 22275381 PMC3335376

[B32] Agirre X Vizmanos JL Calasanz MJ Garcia-Delgado M Larrayoz MJ Novo FJ Methylation of CpG dinucleotides and/or CCWGG motifs at the promoter of TP53 correlates with decreased gene expression in a subset of acute lymphoblastic leukemia patients Oncogene 2003 22 7 1070 1072 10.1038/sj.onc.1206236 12592393

[B33] Valganon M Giraldo P Agirre X Larrayoz MJ Rubio-Martinez A Rubio-Felix D Calasanz MJ Odero MD p53 Aberrations do not predict individual response to fludarabine in patients with B-cell chronic lymphocytic leukaemia in advanced stages Rai III/IV British J Haematology 2005 129 1 53 59 10.1111/j.1365-2141.2005.05405.x 15801955

[B34] Amara K Trimeche M Ziadi S Laatiri A Hachana M Sriha B Mokni M Korbi S Presence of simian virus 40 DNA sequences in diffuse large B-cell lymphomas in Tunisia correlates with aberrant promoter hypermethylation of multiple tumor suppressor genes Int J Cancer 2007 121 12 2693 2702 10.1002/ijc.23038 17724719

[B35] Vilas-Zornoza A Agirre X Martin-Palanco V Martin-Subero JI San Jose-Eneriz E Garate L Alvarez S Miranda E Rodriguez-Otero P Rifon J Torres A Calasanz MJ Cruz Cigudosa J Roman-Gomez J Prosper F Frequent and simultaneous epigenetic inactivation of TP53 pathway genes in acute lymphoblastic leukemia PloS One 2011 6 2 e17012 10.1371/journal.pone.0017012 21386967 PMC3046174

[B36] Fenaux P Mufti GJ Hellstrom-Lindberg E Santini V Finelli C Giagounidis A Schoch R Gattermann N Sanz G List A Gore SD Seymour JF Bennett JM Byrd J Backstrom J Zimmerman L McKenzie D Beach C Silverman LR Efficacy of azacitidine compared with that of conventional care regimens in the treatment of higher-risk myelodysplastic syndromes: a randomised, open-label, phase III study Lancet Oncol 2009 10 3 223 232 10.1016/S1470-2045(09)70003-8 19230772 PMC4086808

[B37] Paulson K Kumar R Ahsanuddin A Seftel MD Azacytidine as a novel agent in the treatment of acute lymphoblastic leukemia Leuk Lymphoma 2011 52 1 134 136 10.3109/10428194.2010.512965 20858102

[B38] Yanez L Bermudez A Richard C Bureo E Iriondo A Successful induction therapy with decitabine in refractory childhood acute lymphoblastic leukemia Leukemia 2009 23 7 1342 1343 10.1038/leu.2009.58 19322208

[B39] Malik A Shoukier M Garcia-Manero G Wierda W Cortes J Bickel S Keating MJ Estrov Z Azacitidine in fludarabine-refractory chronic lymphocytic leukemia: a phase II study Clin Lymphoma Myeloma Leuk 2013 13 3 292 295 10.1016/j.clml.2012.11.009 23265768 PMC3860181

[B40] Blum KA Liu Z Lucas DM Chen P Xie Z Baiocchi R Benson DM Devine SM Jones J Andritsos L Flynn J Plass C Marcucci G Chan KK Grever MR Byrd JC Phase I trial of low dose decitabine targeting DNA hypermethylation in patients with chronic lymphocytic leukaemia and non-Hodgkin lymphoma: dose-limiting myelosuppression without evidence of DNA hypomethylation Br J Haematol 2010 150 2 189 195 20456354 10.1111/j.1365-2141.2010.08213.x PMC2917115

[B41] Norberg M Lindhagen E Kanduri M Rickardson L Sundstrom C Stamatopoulos K Rosenquist R Aleskog A Screening for cytotoxic compounds in poor-prognostic chronic lymphocytic leukemia Anticancer Res 2012 32 8 3125 3136 22843883

[B42] Byrd JC Marcucci G Parthun MR Xiao JJ Klisovic RB Moran M Lin TS Liu S Sklenar AR Davis ME Lucas DM Fischer B Shank R Tejaswi SL Binkley P Wright J Chan KK Grever MR A phase 1 and pharmacodynamic study of depsipeptide (FK228) in chronic lymphocytic leukemia and acute myeloid leukemia Blood 2005 105 3 959 967 15466934 10.1182/blood-2004-05-1693

[B43] Kirschbaum MH Goldman BH Zain JM Cook JR Rimsza LM Forman SJ Fisher RI A phase 2 study of vorinostat for treatment of relapsed or refractory Hodgkin lymphoma: Southwest Oncology Group Study S0517 Leuk Lymphoma 2012 53 2 259 262 10.3109/10428194.2011.608448 21823829 PMC3477846

[B44] Younes A Sureda A Ben-Yehuda D Zinzani PL Ong TC Prince HM Harrison SJ Kirschbaum M Johnston P Gallagher J Le Corre C Shen A Engert A Panobinostat in patients with relapsed/refractory Hodgkin’s lymphoma after autologous stem-cell transplantation: results of a phase II study J Clin Oncol 2012 30 18 2197 2203 10.1200/JCO.2011.38.1350 22547596

[B45] Stumpel DJ Schneider P Seslija L Osaki H Williams O Pieters R Stam RW Connectivity mapping identifies HDAC inhibitors for the treatment of t(4;11)-positive infant acute lymphoblastic leukemia Leukemia 2012 26 4 682 692 10.1038/leu.2011.278 22015773

[B46] Bhatla T Wang J Morrison DJ Raetz EA Burke MJ Brown P Carroll WL Epigenetic reprogramming reverses the relapse-specific gene expression signature and restores chemosensitivity in childhood B-lymphoblastic leukemia Blood 2012 119 22 5201 5210 10.1182/blood-2012-01-401687 22496163 PMC3369610

[B47] Kalac M Scotto L Marchi E Amengual J Seshan VE Bhagat G Ulahannan N Leshchenko VV Temkin AM Parekh S Tycko B O’Connor OA HDAC inhibitors and decitabine are highly synergistic and associated with unique gene-expression and epigenetic profiles in models of DLBCL Blood 2011 118 20 5506 5516 10.1182/blood-2011-02-336891 21772049 PMC3217353

[B48] Nath N Labaze G Rigas B Kashfi K NO-donating aspirin inhibits the growth of leukemic Jurkat cells and modulates beta-catenin expression Biochem Biophys Res Commun 2005 326 1 93 99 15567157 10.1016/j.bbrc.2004.11.009

[B49] Razavi R Gehrke I Gandhirajan RK Poll-Wolbeck SJ Hallek M Kreuzer KA Nitric oxide-donating acetylsalicylic acid induces apoptosis in chronic lymphocytic leukemia cells and shows strong antitumor efficacy in vivo Clin Cancer Res 2011 17 2 286 293 10.1158/1078-0432.CCR-10-1030 21097689

[B50] Park CH Chang JY Hahm ER Park S Kim HK Yang CH Quercetin, a potent inhibitor against beta-catenin/Tcf signaling in SW480 colon cancer cells Biochem Biophys Res Commun 2005 328 1 227 234 10.1016/j.bbrc.2004.12.151 15670774

[B51] Gelebart P Anand M Armanious H Peters AC Dien Bard J Amin HM Lai R Constitutive activation of the Wnt canonical pathway in mantle cell lymphoma Blood 2008 112 13 5171 5179 10.1182/blood-2008-02-139212 18787224 PMC2597612

[B52] Kawahara T Kawaguchi-Ihara N Okuhashi Y Itoh M Nara N Tohda S Cyclopamine and quercetin suppress the growth of leukemia and lymphoma cells Anticancer Res 2009 29 11 4629 4632 20032413

[B53] Gandhirajan RK Staib PA Minke K Gehrke I Plickert G Schlosser A Schmitt EK Hallek M Kreuzer KA Small molecule inhibitors of Wnt/beta-catenin/lef-1 signaling induces apoptosis in chronic lymphocytic leukemia cells in vitro and in vivo Neoplasia 2010 12 4 326 335 20360943 10.1593/neo.91972 PMC2847740

[B54] He B You L Uematsu K Xu Z Lee AY Matsangou M McCormick F Jablons DM A monoclonal antibody against Wnt-1 induces apoptosis in human cancer cells Neoplasia 2004 6 1 7 14 15068666 10.1016/s1476-5586(04)80048-4 PMC1508626

[B55] Mikami I You L He B Xu Z Batra S Lee AY Mazieres J Reguart N Uematsu K Koizumi K Jablons DM Efficacy of Wnt-1 monoclonal antibody in sarcoma cells BMC cancer 2005 5 53 10.1186/1471-2407-5-53 15913453 PMC1164405

[B56] Pode-Shakked N Harari-Steinberg O Haberman-Ziv Y Rom-Gross E Bahar S Omer D Metsuyanim S Buzhor E Jacob-Hirsch J Goldstein RS Mark-Danieli M Dekel B Resistance or sensitivity of Wilms’ tumor to anti-FZD7 antibody highlights the Wnt pathway as a possible therapeutic target Oncogene 2011 30 14 1664 1680 10.1038/onc.2010.549 21472018

[B57] Ettenberg SA Charlat O Daley MP Liu S Vincent KJ Stuart DD Schuller AG Yuan J Ospina B Green J Yu Q Walsh R Li S Schmitz R Heine H Bilic S Ostrom L Mosher R Hartlepp KF Zhu Z Fawell S Yao YM Stover D Finan PM Porter JA Sellers WR Klagge IM Cong F Inhibition of tumorigenesis driven by different Wnt proteins requires blockade of distinct ligand-binding regions by LRP6 antibodies Proc Natl Acad Sci U S A 2010 107 35 15473 15478 10.1073/pnas.1007428107 20713706 PMC2932603

[B58] Lavergne E Hendaoui I Coulouarn C Ribault C Leseur J Eliat PA Mebarki S Corlu A Clement B Musso O Blocking Wnt signaling by SFRP-like molecules inhibits in vivo cell proliferation and tumor growth in cells carrying active beta-catenin Oncogene 2011 30 4 423 433 10.1038/onc.2010.432 20856206 PMC3501789

[B59] Younes A Romaguera J Fanale M McLaughlin P Hagemeister F Copeland A Neelapu S Kwak L Shah J De Castro Faria S Hart S Wood J Jayaraman R Ethirajulu K Zhu J Phase I study of a novel oral janus kinase 2 inhibitor, SB1518, in patients with relapsed lymphoma: evidence of clinical and biologic activity in multiple lymphoma subtypes J Clin Oncol 2012 30 33 4161 4167 10.1200/JCO.2012.42.5223 22965964 PMC5950499

[B60] Yin C Lee S Ayello J van de Ven C Pao J Mulvey E Cairo MS JAK1/JAK2 Inhibitor, ruxolitinib inhibits cell proliferation and induces apoptosis in hodgkin lymphomas (HL) and primary mediastinal B-cell lymphomas (PMBL) ASH Annu Meet Abstr 2012 120 21 4886

[B61] Hart S Goh KC Novotny Diermayr V Hu CY Hentze H Tan YC Madan B Amalini C Loh YK Ong LC William AD Lee A Poulsen A Jayaraman R Ong KH Ethirajulu K Dymock BW Wood JW SB1518, a novel macrocyclic pyrimidine-based JAK2 inhibitor for the treatment of myeloid and lymphoid malignancies Leukemia 2011 25 11 1751 1759 10.1038/leu.2011.148 21691275

[B62] Lanuti P Bertagnolo V Pierdomenico L Bascelli A Santavenere E Alinari L Capitani S Miscia S Marchisio M Enhancement of TRAIL cytotoxicity by AG-490 in human ALL cells is characterized by downregulation of cIAP-1 and cIAP-2 through inhibition of Jak2/Stat3 Cell res 2009 19 9 1079 1089 10.1038/cr.2009.80 19564891

[B63] Diaz T Navarro A Ferrer G Gel B Gaya A Artells R Bellosillo B Garcia-Garcia M Serrano S Martinez A Monzo M Lestaurtinib inhibition of the Jak/STAT signaling pathway in hodgkin lymphoma inhibits proliferation and induces apoptosis PloS one 2011 6 4 e18856 10.1371/journal.pone.0018856 21533094 PMC3080386

[B64] Kim BH Yin CH Guo Q Bach EA Lee H Sandoval C Jayabose S Ulaczyk-Lesanko A Hall DG Baeg GH A small-molecule compound identified through a cell-based screening inhibits JAK/STAT pathway signaling in human cancer cells Mol Cancer Ther 2008 7 9 2672 2680 10.1158/1535-7163.MCT-08-0309 18790749 PMC3646365

