# Supplementary material for: Genetic methylation and lymphoid malignancies: biomarkers of tumor progression and targeted therapy
Source: Biomark Res. 2013 Aug 14;1:24. doi: 10.1186/2050-7771-1-24 (PMC4101819; doi:10.1186/2050-7771-1-24)
Supplement: Additional file 1 — Identified gene hypermethylation in lymphoid malignancies [file 2050-7771-1-24-S1.docx]

**Additional file 1: Table S1. Identified gene hypermethylation in lymphoid malignancies.**

| Disease Type | Methods | Samples | Hypermethylated Genes | Signaling Pathways | Clinical Relevance | Ref |
| --- | --- | --- | --- | --- | --- | --- |
| ALL | MS-PCR | 50 T-ALL patients | *NES-1*, *ADAMTS5*, *WIF-*1, *sFRP-1* | WNT pathway  JAK-STAT pathway  p53 pathway | CIMP-positive patients have shorter DFS and OS compared to CIMP-negative cases | [[9](#_ENREF_9)] |
|  | Genome-wide Infinium HumanMethylation27 BeadChip (27578 CpGs) | 19 B-ALL patients | *MYOD1, PTPRZ1, PPARG, sFRP-1, FOXE3, FBXO39, PKDREJ, TCF3, EGR4, BTG4, PAX5, IKZF1, TLX3, RAG1, POU2AF1* | WNT pathway | All fifteen genes discriminate B-ALL at diagnosis to remission | [[10](#_ENREF_10)] |
|  | Genome-wide custom-designed GoldenGate methylation analysis (1320 CpGs) | 401 ALL patients | *COBL, COL6A2, CPVL, DFNB31, EYA4, FAM24B, FAT1, FUCA2, INADL, MYO3A, PCDHGA12, PON3, ROR1, SYNM, TNIK, ZNF502* | NA | *COBL, CPVL, EVC, LRP1B, PAX8, PCDHGA12* and *SPON2* are correlated with favorable prognosis | [[11](#_ENREF_11)] |
|  | Genome-wide Infinium HumanMethylation27 BeadChip (27578 CpGs) | 33 ALL patients | *CDKN2A, COL6A2, PTPRO*, *CSMD1* | WNT pathway  PI3K pathway | Genomic methylation level higher in relapse than at diagnosis | [[12](#_ENREF_12)] |
| CLL | Genome-wide Infinium HumanMethylation27 BeadChip (27578 CpGs) | 23 CLL patients | *IGHV*-unmutated: *ABI3, SCGB2A1, VHL, GPX3, IGSF4*, *SERPIND5*  *IGHV*-mutated: *ADORA3, AIRE, CARD15, LOC340061, UNC5CL, LDOC1, PRF1, FABP7* | NF-κB pathway  MAPK pathway | Methylation pattern differs between *IGHV*-unmutated and *IGHV*-mutated subgroups | [[15](#_ENREF_15)] |
|  | Genome-wide Methylated CpG island Amplification | 78 CLL patients | *SOX11, DLX1, FAM62C, SOX14, RSPO1, ADCY5, HAND2, SPOCK, MLL, ING1, PRIMA1, BCL11B, LTBP2, BNC1, NR2F2, SALL1, GALGT2, LHX1, DLX4, KLK10, TFAP2, APP* | p53 pathway  Notch pathway | *LINE, APP, SALL1* and *PRIMA1* are correlated with shorter OS | [[16](#_ENREF_16)] |
| DLBCL | Genome-wide CpG island microarray assay (500 CpGs) | 21 ABC-DLBCL 24 GCB-DLBCL patients | *FLJ21062, BNIP3, MGMT, RBP1, GATA4, IGSF4*, *CRABP1* | Rho pathway | *FLJ21062*, *ONECUT2* and *GNMT* methylation differs between GCB-DLBCL and ABC-DLBCL | [[18](#_ENREF_18)] |
|  | *HpaII* tiny fragment Enrichment by Ligation-mediated PCR (HELP) assay | 69 DLBCL patients | *LANCL1, KCNK12, SORL1, CXorf57, SOX9, KIAA0746, ASPHD2, ARHGAP17, IKZF1, PMM2, IL12A, JDP2, PAK1, GALNS, FGD2*, *LYAR* | JAK-STAT pathway | All sixteen genes distinguish GCB-DLBCL and ABC-DLBCL | [[19](#_ENREF_19)] |
| MCL | MassARRAY EpiTYPERH assay | 38 MCL patients  7 MCL cell lines | *SOX9*, *HOXA9*, *AHR*, *NR2F2*, *ROBO1*, *NPTX2*, *CDH1* | p53 pathway | Patients with two or more methylated genes have higher Ki-67 index and shorter OS | [[20](#_ENREF_20)] |
|  | HELP assay (50000 CpGs) | 22 MCL patients  10 controls | *CDKN2B, HOXD8, MLF-1*, *PCDH8* | NA | NA | [[21](#_ENREF_21)] |
| HL | Methylation specific-PCR and Bisulfite Genomic sequencing | 7 HL cell lines  40 HL patients | *IGSF4, CD44*, *GADD45*, *ZMAT3*, *IRF7, KLF6* | p53 pathway | NA | [[22](#_ENREF_22)] |
|  | Genome-wide 27K BeadArray analysis (24312 CpGs) | 5 HL cell lines | 329 commonly hypermethylated genes | WNT pathway | NA | [[23](#_ENREF_23)] |

ALL: acute lymphoblastic leukemia; MS-PCR: methylation specific-polymerase chain reaction; CIMP: CpG island methylator phenotype; DFS: disease-free survival; OS: overall survival; NA: not available; CLL: chronic lymphocytic leukemia; *IGHV*: immunoglobulin heavy-chain variable; DLBCL: diffuse large B-cell lymphoma; ABC: activated B-cell-like; GCB: germinal center B-cell-like; HELP: *HpaII* tiny fragment Enrichment by Ligation-mediated PCR; MCL: mantle cell lymphoma; HL: Hodgkin’s lymphoma; *ABI3*: ABI family, member 3; *ADAMTS5*: A disintegrin and metalloproteinase with thrombospondin motifs 5; *ADCY5*: adenylate cyclase 5; *ADORA3*: adenosine A3 receptor; *AHR*: aryl hydrocarbon receptor; *AIRE*: autoimmune regulator; *APP*: amyloid beta (A4) precursor protein; *ARHGAP17*: Rho GTPase activating protein 17; *ASPHD2*: aspartate beta-hydroxylase domain containing 2; *BCL11B*: B-cell CLL/lymphoma 11B; *BNC1*: basonuclin 1; *BNIP3*: BCL2/adenovirus E1B 19kDa interacting protein 3; *BTG4*: B-cell translocation gene 4; *CARD15*: caspase recruitment domain family, member 15; *CDH1*: cadherin 1, type 1; *CDKN2A*: cyclin-dependent kinase inhibitor 2A; *CDKN2B*: cyclin-dependent kinase inhibitor 2B; *COBL*: cordon-bleu WH2 repeat protein; *COL6A2*: collagen, type VI, alpha 2; *CPVL*: carboxypeptidase, vitellogenic-like; *CRABP1*: cellular retinoic acid binding protein 1; *CSMD1*: CUB and Sushi multiple domains 1; *CXorf57*: chromosome X open reading frame 57; *DFNB31*: deafness, autosomal recessive 31; *DLX1*: distal-less homeobox 1; *DLX4*: distal-less homeobox 4; *EGR4*: early growth response protein 4; *EVC*: Ellis van Creveld syndrome; *EYA4*: eyes absent homolog 4; *FABP7*: fatty acid binding protein 7, brain; *FAM24B*: family with sequence similarity 24, member B; *FAM62C*: family with sequence similarity 62 (C2 domain containing), member C; *FAT1*: FAT atypical cadherin 1; *FBXO39*: F-box only protein 39; *FOXE3*: forkhead box protein E3; *FUCA2*: fucosidase, alpha-L- 2, plasma; *FGD2*: FGD1 family, member 2; *GADD45*: growth arrest and DNA-damage-inducible 45 alpha; *GALGT2*: beta-1,4-N-acetyl-galactosaminyl transferase 2; *GALNS*: galactosamine (N-acetyl)-6-sulfate sulfatase; *GATA4*: GATA binding protein 4; *GNMT*: glycine N-methyltransferase; *GPX3:* glutathione peroxidase 3 (plasma); *HAND2*: heart and neural crest derivatives expressed 2; *HOXA9*: homeobox A9; *HOXD8*: homeobox D8; *IGSF4*: immunoglobulin superfamily member 4; *IKZF1*: IKAROS family zinc finger 1; *IL12A*: interleukin 12A; *INADL*: inactivation no after-potential D-like protein; *ING1*: inhibitor of growth family, member 1; *IRF7*: interferon regulatory factor 7; *JDP2*: Jun dimerization protein 2; *KCNK12*: potassium channel, subfamily K, member 12; *KIAA0746*: sel-1 suppressor of lin-12-like 3; *KLF6*: Kruppel-like factor 6; *KLK10*: kallikrein-related peptidase 10; *LANCL1*: LanC lantibiotic synthetase component C-like 1; *LDOC1*: leucine zipper, down-regulated in cancer 1; *LHX1*: LIM homeobox 1; *LRP1B*: low density lipoprotein receptor-related protein 1B; *LTBP2*: latent transforming growth factor beta binding protein 2; *LYAR*: Ly1 antibody reactive; *MGMT*: methylguanine-DNA methyltransferase; *MLF-1*: myeloid leukemia factor 1; *MLL*: myeloid/lymphoid or mixed-lineage leukemia; *MYOD1*: myogenic differentiation 1; *MYO3A*: myosin IIIA; *NES-1*: normal epithelial cell-specific 1; *NPTX2*: neuronal pentraxin II; *NR2F2*: nuclear receptor subfamily 2, group F, member 2; *ONECUT2*: one cut homeobox 2; *PAK1*: p21 protein (Cdc42/Rac)-activated kinase 1; *PAX5*: paired box 5; *PAX8*: paired box 8; *PCDHGA12*: protocadherin gamma subfamily A, 12; *PCDH8*: protocadherin 8; *PKDREJ*: polycystic kidney disease and receptor for egg jelly-related protein; *PMM2*: phosphomannomutase 2; *PON3*: paraoxonase 3; *POU2AF1*: POU class 2 associating factor 1; *PPARG*: peroxisome proliferator-activated nuclear receptor gamma; *PRF1*: perforin 1; *PRIMA1*: proline rich membrane anchor 1; *PTPRO*: protein tyrosine phosphatase, receptor type, O; *PTPRZ1*: protein tyrosine phosphatase, receptor-type, zeta polypeptide 1; *RAG1*: recombination activating gene 1; *RBP1*: retinol binding protein 1, cellular; *ROBO1*: roundabout, axon guidance receptor, homolog 1; *ROR1*: receptor tyrosine kinase-like orphan receptor 1; *RSPO1*: R-spondin 1; *SALL1*: sal-like 1; *SCGB2A1*: secretoglobin, family 2A, member 1; *sFRP-1*: secreted frizzled-related protein 1; *SORL1*: sortilin-related receptor, L(DLR class) A repeats containing; *SOX9*: SRY (sex determining region Y)-box 9; *SOX11*: SRY (sex determining region Y)-box 11; *SOX14*: SRY (sex determining region Y)-box 14; *SPOCK*: sparc/osteonectin, cwcv and kazal-like domains proteoglycan (testican) 1; *SPON2*: spondin 2, extracellular matrix protein; *SYNM*: synemin, intermediate filament protein; *TCF3*: transcription factor 3; *TFAP2*: transcription factor AP-2 alpha; *TLX3*: T-cell leukemia homeobox 3; *TNIK*: TRAF2 and NCK interacting kinase; *UNC5CL*: unc-5 homolog C –like; *VHL*: von Hippel-Lindau tumor suppressor; *WIF-1*: wnt inhibitory factor 1; *ZMAT3*: zinc finger, matrin-type 3; *ZNF502*: zinc finger protein 502.
